# Cost-Effectiveness Analysis of PEG-rhG-CSF as Primary Prophylaxis to Chemotherapy-Induced Neutropenia in Women With Breast Cancer in China: Results Based on Real-World Data

**DOI:** 10.3389/fphar.2021.754366

**Published:** 2022-02-03

**Authors:** Jie Zhao, Gaoxing Qiao, Yan Liang, Jia Li, Wei Hu, Xu Zuo, Junfang Li, Chenglong Zhao, Xiaojian Zhang, Shuzhang Du

**Affiliations:** ^1^ Department of Pharmacy, The First Affiliated Hospital of Zhengzhou University, Zhengzhou, China; ^2^ Department of Pharmacy, Xinyang Central Hospital, Xinyang, China; ^3^ Department of Pharmacy, The Affiliated People’s Hospital of Xinxiang Medical University, Xinxiang, China; ^4^ Department of Pharmacy, The First Affiliated Hospital of Henan University of Science and Technology, Luoyang, China; ^5^ Department of Pharmacy, Henan Provincial People’s Hospital, Zhengzhou, China

**Keywords:** cost-effectiveness, PEG-rhG-CSF, chemotherapy-induced neutropenia, breast cancer, real-world

## Abstract

**Background:** Pegylated recombinant human granulocyte colony-stimulating factors (PEG-rhG-CSFs) are more commonly and widely used than recombinant human granulocyte colony-stimulating factors (rhG-CSFs) in preventing chemotherapy-induced neutropenia in patients with stage II-IV breast cancer. To reduce the financial burden on these patients, the corresponding medical insurance directory needs to be revised.

**Objectives:** To evaluate the cost-effectiveness of PEG-rhG-CSF versus rhG-CSF in patients with stage II-IV breast cancer in central China.

**Methods:** Two Markov models, a chemotherapy model and a post-chemotherapy model, were developed to study the effects and costs, with a time horizon of 12 weeks and 35 years, respectively. Cost and probability input data were primarily obtained from a retrospective real-world study conducted in five tertiary hospitals. Propensity score matching was adopted to overcome retrospective bias. Other parameters were extracted from literature as well as advice from clinical experts. Univariate and probabilistic sensitivity analyses were conducted.

**Results:** In the first chemotherapy model, PEG-rhG-CSF was associated with fewer episodes of febrile neutropenia (FN) (N = 19 per 1000 patients treated), infections (N = 24 per 1000 patients treated) and deaths (N = 2 per 1000 patients treated), but higher costs (¥36 more per patient treated). The post-chemotherapy model indicated that PEG-rhG-CSF led to higher gains in quality-adjusted life years (QALYs) (11.695 versus 11.516) in comparison to rhG-CSF. Sensitivity analysis showed that the cost of PEG-rhG-CSF had the greatest impact on the incremental costs, and incremental QALYs were very sensitive to the risk of RDI <85%. The probability of PEG-rhG-CSF being cost-effective compared to rhG-CSF was 66% at the willingness to pay (WTP) thresholds of ¥72,371 per QALY gained.

**Conclusion:** According to this economic evaluation based on real-world data, PEG-rhG-CSF may be considered as a more cost-effective strategy relative to rhG-CSF for stage II-IV breast cancer patients in central China. However, to reflect a national perspective, further evidence is needed using data from larger-scale studies.

## 1 Introduction

The incidence of breast cancer tops the female cancers in China, and the age standardization incidence rate (ASIR) is increasing every year (World Health Organization, 2020). In 2018, 98000 women died of breast cancer in China, accounting for 15% of all cancer-related deaths in women (Bray et al., 2018). In the era of precision medicine, chemotherapy remains the cornerstone of treatment for patients with breast cancer (Chinese Society of Clinical Oncology Guidance Working Committee, 2017), not only because adjuvant chemotherapy significantly improves disease-free and overall survival, but also due to the chemotherapy directly leads to improved patient survival (Esteva et al., 2001; Peto et al., 2012). Accompanying the chemotherapy, however, neutropenia is a common and frequent side effect, as well as a major risk factor for infection-related morbidity and mortality (Donadieu et al., 2011). Prolonged and severe neutropenia may lead to serious toxicity such as febrile neutropenia (FN). The presence of FN in cancer patients may lead to reduced dose intensity (RDI), worsening clinical efficacy, as well as severe infection complications, even death (Chinese Society of Clinical Oncology Guidance Working Committee, 2017). Under the current medical conditions, when the patient’s neutropenia lasts for >21 days, the incidence of infection is significantly increased (Chinese Society of Hematology, 2020). Consequently, the patient’s quality of life is affected, and the clinical efficacy and cost-effectiveness of chemotherapy may be compromised (Lathia et al., 2013).

To counteract the negative impact of neutropenia, short and long acting granulocyte-colony stimulating factors (G-CSFs) are used to enhance the proliferation, differentiation, and maturation of neutrophils (Knudsen et al., 2011), thereby reducing the duration and severity of neutropenia, as well as the incidence of FN and infection-related mortality (Kuderer et al., 2007; Wang et al., 2015). The Chinese Society of Clinical Oncology recommends using G-CSFs as primary prophylaxis with chemotherapy regimens associated with a ≥20% incidence of FN (Chinese Society of Clinical Oncology Guidance Working Committee, 2017). Some randomized controlled trials (RCTs) in China also demonstrated that both short and long acting G-CSFs showed equal reduction in the incidence of FN (Sheng et al., 2015; Jiang et al., 2018; Xie et al., 2018; Liu et al., 2019), although there is no economic evidence. Currently, long-acting G-CSFs (PEG-rhG-CSFs) are more often used than short-acting G-CSFs (rhG-CSFs).

Although several cost-effectiveness analyses evaluating G-CSFs have been published (Akpo et al., 2017; Gao and Li, 2018; Li-Tian et al., 2019; Xia et al., 2020), all of them assumed a cost-effective benefit associated with PEG-rhG-CSFs based on RCTs.

The objective of this study was to determine whether primary prophylaxis against FN and related infections using either PEG-rhG-CSFs or rhG-CSFs in female breast cancer patients undergoing a four-cycle TC (docetaxel and cyclophosphamide) chemotherapy is cost-effective from a real-world setting.

## 2 Materials and Methods

### Model Design

A mathematical model was developed in Excel (Microsoft 2016) to estimate the health benefits and costs of using PEG-rhG-CSF compared with rhG-CSF as the primary prophylaxis in two hypothetical cohorts of women with stage II, III, IV breast cancer undergoing chemotherapy. Two Markov models were generated, one tracked on-chemotherapy cycles and neutropenia-related complications (FN and infection) (model 1) and another captured the impact of RDI on long-term survival (model 2). All patients entered the model at the average age of 45 years, and in the state labeled “chemotherapy” upon administration of chemotherapy agents, and G-CSFs (PEG-rhG-CSF or rhG-CSF) on day 2 of each chemotherapy cycle. The costs of treatment were the actual charges of medical services, and were estimated from the Chinese healthcare system perspective, reported in 2019 in Chinese yuan. Based on transition probabilities, the patients either moved to chemotherapy-related complication health states or remained in their current health state.

For the first model, a 3-week cycle length was defined for each of the four chemotherapy cycles, the time horizon in the chemotherapy model was 12 weeks accordingly, deaths associated with FN and infections were considered. No discounting was applied in this model. For the long-term survival model, the annual cycle was taken with a time horizon of 35 years, as the average life expectancy among the Chinese (including the breast cancer patients) is nearly 80 years, and the average age of the patients in our study was 45 years. In accordance with pharmacoeconomic evaluation guidelines (Liu, 2020), both costs and utilities were discounted at 5% each year.

**FIGURE 1:**
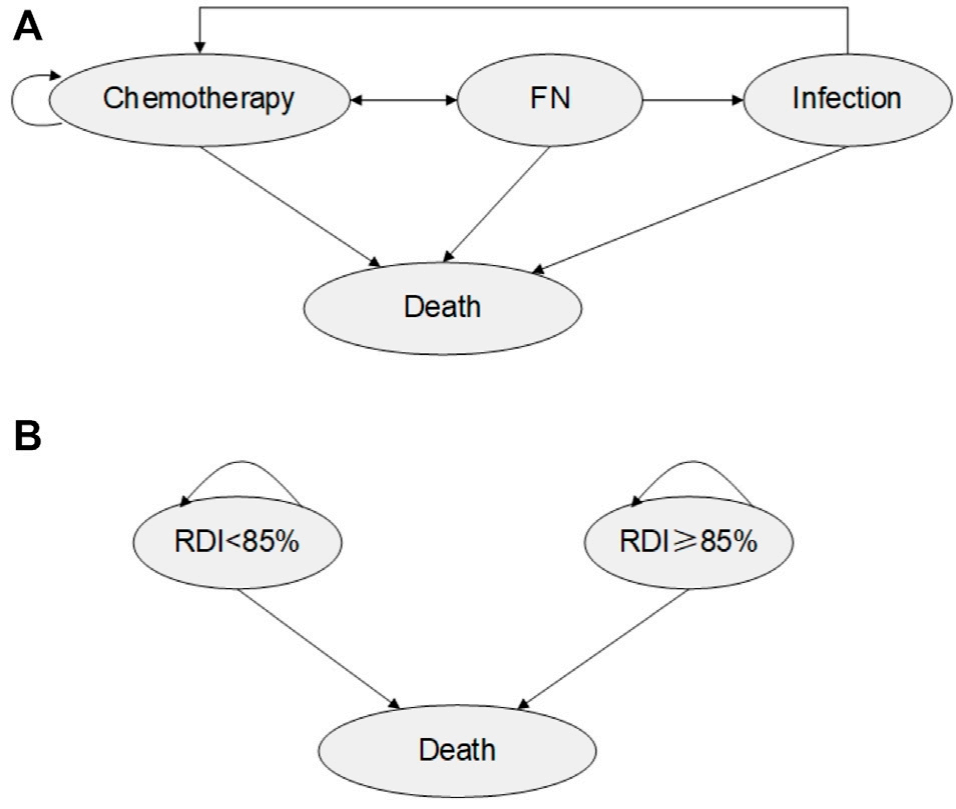
Model Structure **(A)** Model 1: Chemotherapy Markov Model. **(B)** Model 2: Post-chemotherapy Markov model.

Costs and clinical data were obtained through real-world, expert consultation and literature review. The two model structures are shown in Figure 1.

### Real-World Data Collection

Clinical and cost data were obtained through a non-interventional, retrospective observational study of female breast cancer patients from five sites in Henan province, which have the largest population of breast cancer patients in China, and represent the characteristics of the target population of this study. As per the objective of the study, patients below 18 years of age and those with other cancers or underwent both G-CSFs were excluded.

Clinical data were collected from hospital administrative records, by retrospectively including all cases receiving rhG-CSF and a larger sample of patients receiving PEG-rhG-CSF, between January 2019 and December 2020. Patient-level data were de-identified to protect the privacy and sensitive information. Cost data included the total direct medical costs during the hospitalization for chemotherapy, including fees for drugs, examinations, tests, hospitalization, nursing, etc.

Propensity score matching (PSM) was conducted to overcome retrospective bias, by considering age, gender, type of health insurance, and the number of concomitant diseases. In this study, a 1:1 ratio between matched subjects was used. The means of propensity scores after matching were 0.52 for the PEG-rhG-CSF group and 0.49 for the rhG-CSF group. Meanwhile, the results of the data analysis such as mean age and mean costs after PSM adjustment were used as key inputs in the Markov model.

### Model Inputs

#### 2.1.1 Clinical Data

In the chemotherapy model, the main clinical input data was the incidence of FN from the collected real-world patient-level data. The risk of infection in the case of FN was estimated based on the Chinese guidelines for the clinical application of antibacterial drugs for agranulocytosis with fever (2020) (Chinese Society of Hematology, 2020). Some other inputs, utility data, and death rate were obtained through literature review. Additionally, because only limited data were obtainable from real-world data and local literature, eight expert oncologists were consulted to close the data gaps, especially for the cost of infection treatment. These experts were selected based on the hospital category (including general, oncology, and women’s hospitals) and their experience with breast cancer. Table 1 summarizes the parameter values and their sources.

**TABLE 1:** Summary of input parameters for the chemotherapy model.

Parameter	Base case value		Distribution for PSA	Source^a^
	PEG-rhG-CSF	rhG-CSF		
Transition probabilities				
Baseline of FN event across all chemotherapy cycles	0.0116	0.0404	Beta	A
Risk of infection in patients with FN	0.0547	0.547	Beta	B
Risk of death in patients with FN	0.034	0.034	Beta	Xia et al.
Risk of death if infection	0.034	0.034	Beta	C
Cost inputs (¥)				
G-CSF, per cycle	3315.74	734.34 (6d)	Gamma	A
Chemotherapy, per cycle				
Docetaxel	1792.74 (20mg/0.5 ml)		Gamma	A
Cyclophosphamide	120.75 (0.2g)		Gamma	A
FN inpatient, per patient	25000		Gamma	C
Infection if FN, per patient	50000		Gamma	C
Hospitalization(mean)	14811.10		Gamma	D
Utility inputs				
Chemotherapy	0.70		Beta	Akpo et al.
FN inpatient	0.33		Beta	Akpo et al.
Infection	0.33		Beta	Akpo et al.

G-CSF, granulocyte colony-stimulating factor; PSA, probabilistic sensitivity analysis; SA, sensitivity analysis.

a A, real-world data; B, Chinese guidelines for the clinical application of antibacterial drugs for agranulocytosis with fever (2020); C, expert opinion; D, national data of health care from NBS (National Bureau of Statistics of China).

The post-chemotherapy model mainly considered the impact of decreases in RDI on survival. As mentioned above, age and FN event as predictors of receiving RDI <85%. The risk of RDI <85% for the history of FN was calculated from the real-world data, and other model inputs were extracted from literatures. Breast cancer-specific mortality data by stage and age were accessed from the Chinese Cancer Registry Annual Report and all-cause mortality data from the National Bureau of Statistics of China. Table 2 presents the list of parameters used in the post-chemotherapy model.

**TABLE 2:** Summary of input parameters for the post-chemotherapy model.

Parameter	Base case value	Distribution for PSA	Source^a^
Risk of RDI<85% if FN	0.500	Beta (α,β = 191)	A
Risk of RDI<85%, age<65 years old, no FN	0.247	Beta (*α* = 289, *β* = 881)	Akpo et al.
RR of RDI<85% for age≥65 vs. <65 years old	1.380		Akpo et al.
OR of RDI<85%, FN vs. no FN	1.580		Akpo et al.
HR of survival associated with an RDI<85% vs. RDI≥85%	1.730		Gao et al.
Utility of breast cancer in years 1–5	0.860	Beta (*α* = 40, *β* = 6)	Akpo et al.
Utility of breast cancer in years >5	0.960	Beta (*α* = 367, *β* = 15)	Akpo et al.

a A, real-world data.

#### 2.1.2 Costs

The unit cost for resource use related to G-CSFs, chemotherapy regimen, antibiotics/anti-fungals, and average hospitalization cost (including nursing, oncology ward, laboratory tests, examinations, etc.) were considered. Drug cost data (in 2019 CNY-¥) were derived from the local medical procurement platform, considering the average of the list price. Chemotherapy [docetaxel, 75 mg/m2 of body surface area (BSA), plus cyclophosphamide, 600 mg/m2] was administered every 21 days for four cycles. The expert consultation yielded data about the use of antimicrobials for the infections following FN, mainly bacteremia, gastrointestinal, urinary, cellulitis, and fungal infections (Dan-Li et al., 2015), which were estimated as a weighted average of the cost per treatment course, considering relative market share. Only inpatient costs were considered for FN. Simultaneously, it was assumed that the cost of FN and infection hospitalization did not differ between the two G-CSFs. The per cycle cost of hospitalization was obtained from the China Health Statistics Yearbook (2020), considering the mean of all listed medication costs. There were no costs imputed for the post-chemotherapy model. Table 1 summarizes the unit cost populated in the first model.

#### 2.1.3 Utilities

Utility levels for each health state in the chemotherapy and post-chemotherapy models were taken from the published literature (Tables 1, 2). The estimated utility for the state of chemotherapy, FN/infection, breast cancer survivor during years one to five and after year five was 0.7, 0.33, 0.86, and 0.96 (Akpo et al., 2017). As a result of the lack of data on utility values for infection, these data were assumed to be equal to that of FN, according to Gao and Li (2018).

### Sensitivity Analysis

Sensitivity analyses comprised univariate and probabilistic sensitivity analyses. One-way sensitivity analysis was adopted to test the variance of underlying parameter values and assumptions within the models. The variance of each parameter was set to either 95% confidence intervals (CI), where data were available or varied by 15% (according to literature) except for the discount rate, which was set as 3 and 7% (Liu, 2020).

Probabilistic sensitivity analysis (PSA) was conducted with a Monte-Carlo simulation, and all the input parameters on cost-effectiveness outcomes were incorporated into the analysis. Beta and gamma distributions were assigned to each relevant parameter, respectively. One thousand Monte-Carlo simulations were conducted with the value of model inputs randomly drawn from parameter distributions. A cost-effectiveness acceptability curve (CEAC) was presented to show the cost-effectiveness probability of PEG-rhG-CSF for different levels of WTP per QALY gained.

## 3 Results

### Real-World Data

Patient-level real-world data were retrospectively collected from a sample of 926 patients receiving PEG-rhG-CSF and 898 patients receiving rhG-CSF in the selected hospitals. In this primary cohort, the average length of hospital stay in the long-acting (PEG-rhG-CSF) group was 2 days more than in the short-acting (rhG-CSF) group (mean 10.47 ± 7.47 days in the long-acting group and 8.95 ± 7.88 days in the short-acting group; *p* < 0.01), and was associated with more total costs per hospitalization (mean ¥17,079 ± ¥3,084 in the long-acting group and ¥14,086 ± ¥335 in the short-acting group; *p* < 0.01). Meanwhile, surgical rates were also slightly higher in the long-acting group (52.9% in the long-acting group and 40% in the short-acting group). No significant difference was observed in age (mean 48.80 ± 9.56 years in the long-acting group and 48.75 ± 9.96 years in the short-acting group), occupation (most were retirees) and health insurance type (most were urban and rural residents).

PSM resulted in the inclusion of 852 patients each in the intervention and comparator groups. The baseline characteristics were balanced after PSM adjustment, with no significant differences in age, marriage, occupation, insurance type, and the surgery rate between the two groups. The average length of hospital stay in the long-acting group was also 2 days more than in the short-acting group. The total cost for single hospitalization was lower (mean ¥15,909 ± ¥4,960 in the long-acting group and ¥13,097 ± ¥2,968 in the short-acting group).

### Base-Case Results

The cost-effectiveness results of primary prophylaxis with PEG-rhG-CSF compared to rhG-CSF for patients with stage Ⅱ-Ⅳ breast cancer are presented in Table 3. Compared to rhG-CSF, treatment with PEG-rhG-CSF was associated with higher costs (¥36) and higher benefits, that included increased QALYs gained (0.104), and fewer cases of FN (19 vs. 61 per 1000 patients treated), infections (24 vs. 83 per 1000 patients treated) and deaths (2 vs. 8 per 1000 patients treated) in the chemotherapy model. Table 3 also summarizes the effectiveness results from the post-chemotherapy model, which were estimated by a Monte Carlo simulation with 1000 iterations. Over the 35-year time horizon, administration of PEG-rhG-CSF was correlated with slightly higher gains in QALYs (11.695 vs 11.516) than rhG-CSF.

**TABLE 3:** Cost-effectiveness analysis results.

Strategy	Costs, CNY¥	QALYs	Incremental cost, CNY¥	Incremental QALYs	ICER, CNY¥/QALY
Chemotherapy model					
PEG-rhG-CSF	146091	3.456	36	0.104	347
rhG-CSF	146055	3.352	—	—	—
Post-chemotherapy model					
PEG-rhG-CSF		11.695		0.179	
rhG-CSF		11.516		—	

ICER, Incremental cost-effectiveness ratio; QALYs, quality-adjusted life years.

### Sensitivity Analysis Results

#### 3.1.1 Deterministic Sensitivity Analysis

**FIGURE 2:**
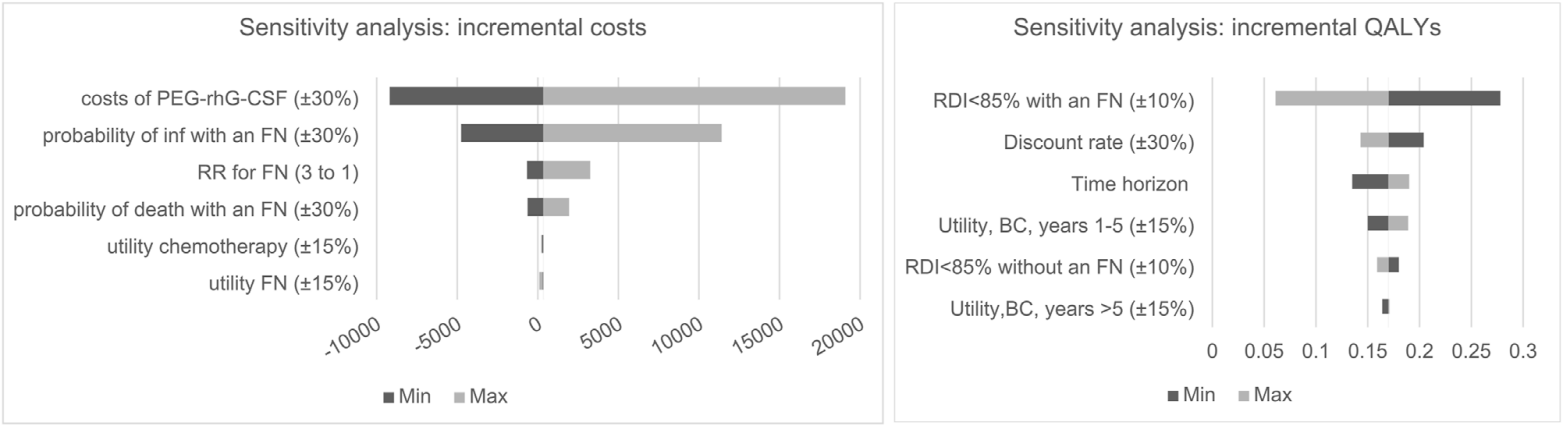
One-way sensitivity analysis tornado diagram for incremental cost and QALY, BC, Breast cancer, inf, infection; FN, ferbrile eutropenia.

One-way sensitivity analysis showed the impact of each model parameter on incremental costs and QALYs, as two tornado diagrams in Figure 2. For the scenarios within the possible ranges of model inputs, increasing FN and infection transition probabilities (30%) made PEG-rhG-CSF less costly compared to rhG-CSF. The cost of PEG-rhG-CSF had the greatest impact on the incremental costs, followed by the risk of infection in patients with FN and the risk of FN following chemotherapy. Incremental QALYs were most sensitive to variance in risk of RDI <85% with an FN. Additionally, QALYs gained decreased as the discount rate increased; and increased as the time horizon extended. Furthermore, QALYs gained increased as the utility for cancer survivors between one to 5 years increased.

#### 3.1.2 Probabilistic Sensitivity Analysis

**FIGURE 3:**
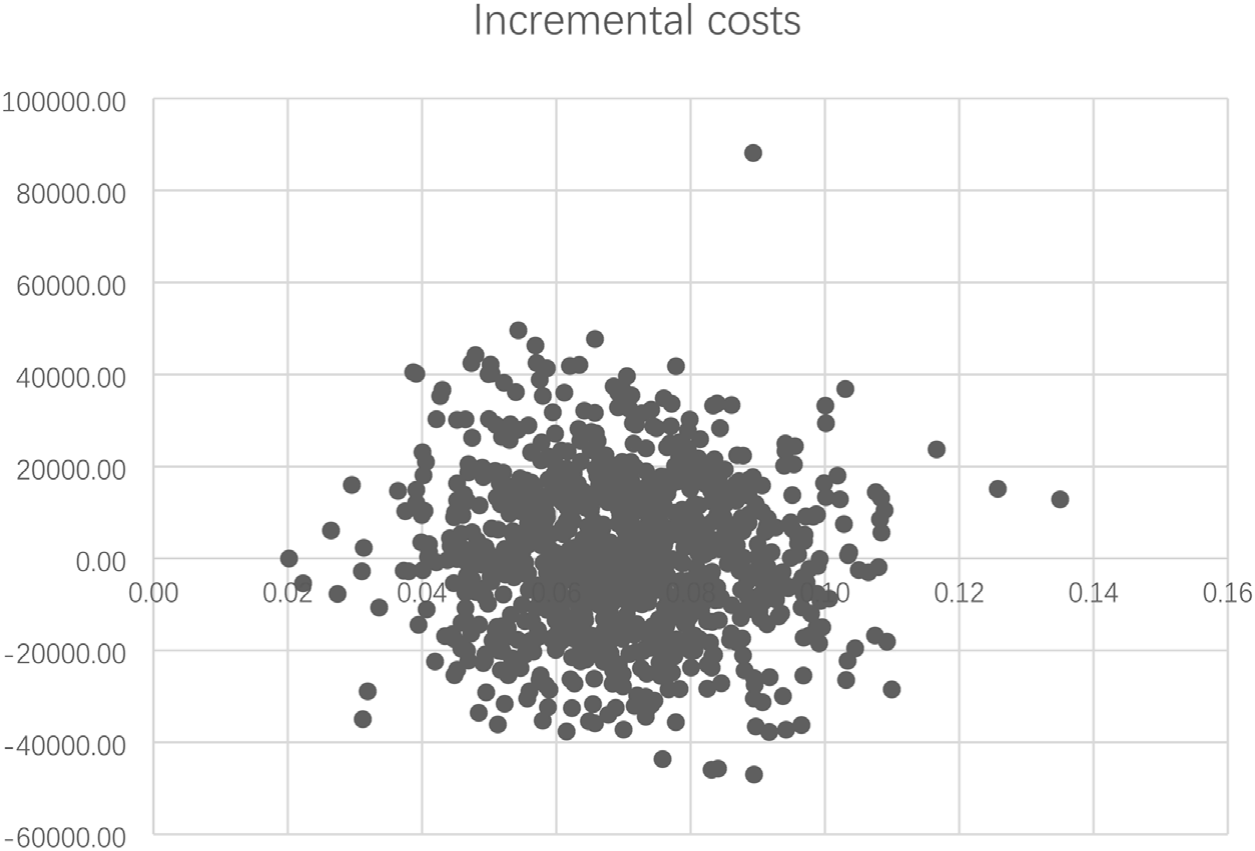
Cost effectiveness plane for PEG-rhg-CSF compared to rhG-CSF.

The PSA results are summarized as a scatterplot in Figure 3, which demonstrated a consistent finding of slightly better QALYs and higher costs for PEG-rhG-CSF in the majority of scenarios. The cost-effectiveness acceptability curve showed that at WTP of ¥72,371 per QALY (2020 GDP per capita to China), the probability that PEG-rhG-CSF would be considered a cost-effective alternative to rhG-CSF was 66% (Supplementary Figure S1).

In addition, the PSA for the post-chemotherapy model showed that administration of PEG-rhG-CSF led to greater gains in QALYs compared to rhG-CSF (PSA results of both models are listed in Supplementary Table S1).

## 4 Discussion

The two G-CSFs compared in the present analysis were manufactured by Chinese pharmaceutical companies, and some clinical evidence demonstrated differences in effectiveness and safety (Liverani et al., 2014; Zhang et al., 2018a; Huang et al., 2018; Bongiovanni et al., 2019; Zhu et al., 2019; Ma et al., 2020; Huang et al., 2021). The PEG-rhG-CSF is recommended as a higher compliance treatment by current guidelines (Chinese Society of Clinical Oncology Guidance Working Committee, 2017). Nonetheless, real-world evidence and economic analysis results are increasingly recognized as an important and reasonable guide for reimbursement decision making in China since the 2017 national pricing negotiation on innovative medicines (Ming et al., 2019). Hence, this study evaluated the cost-effectiveness of PEG-rhG-CSF compared to rhG-CSF based on real-world data in China, with a particular focus on the incidence of FN, infections and RDI <85%. Additionally, QALYs gained were captured as standard measures of effect.

In our simulation modeling study, which applied PSM to real-world data, PEG-rhG-CSF was slightly inferior to rhG-CSF in terms of decreasing the risk of FN. This finding was consistent with published clinical studies (Garcia-Carbonero et al., 2001; Kuderer et al., 2007; Mhaskar et al., 2014). Moreover, the baseline characteristics of the cohort were similar to a previous multi-center randomized controlled phase Ⅳ clinical study in terms of age and chemotherapy regimens, and the conclusion was in line with the risk of FN (Jiang et al., 2018). There were four other health economic analyses in China based on clinical observations or randomized trials (Zhang et al., 2018b; Li-Tian et al., 2019; Xia et al., 2020; Zhang et al., 2020), and the findings were associated with a similar incidence of FN in the PEG-rhG-CSF group, while two of them showed different conclusions. One was using imported medicine (the price was much higher than the domestic drug) as the control group and the other came from a single center with small sample size (Li-Tian et al., 2019; Zhang et al., 2020).

Our analysis demonstrated that PEG-rhG-CSF was more cost-effective compared to rhG-CSF as primary prophylaxis under the WTP threshold of one-time GDP per capita. The result was similar to Xia et al. (2020), but different from Akpo et al. (2017), Gao and Li (2018), and Li-Tian et al. (2019), whose results showed that PEG-rhG-CSF strategy was cost-saving than rhG-CSF. Compared to randomized trials, our study showed that the incidence of FN for the short-acting group was higher than these studies, and the cost of the long-acting group was in excess of approximately ¥2,500, but the price gap in Akpo et al. (2017) and Gao and Li (2018) was zero. This may be one of the reasons why their results differed from ours.

Detailed sensitivity analyses of the key related parameters were performed to test the robustness of the cost-effectiveness conclusion. The base case analysis revealed that cost-savings were maximally influenced by the variation in the cost of PEG-rhG-CSF. As the average unit price of PEG-rhG-CSF is almost 15 times that of rhG-CSF in the current market, the analysis indicated that reducing the unit price of PEG-rhG-CSF by 30% would be cost-saving and dominant on ICER compared to the current price. Effectiveness results were mainly influenced by risk of RDI <85% with an FN, which was in line with Li-Tian et al. (2019) and Xia et al. (2020) who reported the parameter as a key driver of the cost-effectiveness for preventing FN after chemotherapy.

Furthermore, over a 35-year time horizon, PEG-rhG-CSF was likely (66%) to be associated with greater QALYs gained compared to rhG-CSF. Currently, oncology providers and pharmacists have more confidence in improving the usage of CSFs (Wong et al., 2020; Lapidari et al., 2021), and the short-acting agent is often used in patients with acute illness (Zhang et al., 2018b; Ma et al., 2020). Meanwhile, the experts we consulted indicated that the rhG-CSF is currently mainly used for emergency relief and short-term inpatients, and the probability of adoption is decreasing. Since both G-CSFs are covered by Chinese medical insurance, the rhG-CSF could be withdrawn from the health insurance directory to benefit a wider population of patients.

The strengths of this study include the use of data from real-world settings. The real-world data-based cost-effectiveness analysis clarified the impact of FN and related risk factors on its severity, as well as treatment effectiveness and economic impact of the management of neutropenia. These findings will be helpful in policymaking and health resource-planning.

This study had several limitations. First, the real-world data collected was from retrospective sources, due to reliance upon electronic health records, which could be less reliable than a prospective study (Ming et al., 2019), even though the PSM was adopted to surmount the potential bias. Second, the transition probabilities populated in each model were derived from limited empirical data source, some critical parameters and utility values were obtained from recommendations of the advisory group and international studies. Although we performed sensitivity analysis for the related parameters, the bias borne by this uncertainty might be minimized. Third, the post-chemotherapy costs were assumed to be zero according to the cost of G-CSFs, and associated costs were captured in the chemotherapy model. In addition, there is limited data from electronic records on the impact of RDI on resource utilization and related costs, as long-term costs cannot be accurately estimated. As the heterogeneous array of population were only Chinese, different geographic variations and ethnic groups can be included in further analysis.

In summary, this real-world data-based health economic evaluation showed that, comparing with rhG-CSF, PEG-rhG-CSF may be more cost-effective for the management of patients with stage Ⅱ-Ⅳ breast cancer in the central region of China. Further data from national wide may be needed for a more comprehensive analysis.

## Data Availability

The original contributions presented in the study are included in the article/Supplementary Material, further inquiries can be directed to the corresponding authors.
