# Supplementary material for: Cost-Effectiveness Analysis of PEG-rhG-CSF as Primary Prophylaxis to Chemotherapy-Induced Neutropenia in Women With Breast Cancer in China: Results Based on Real-World Data
Source: Front Pharmacol. 2022 Feb 3;12:754366. doi: 10.3389/fphar.2021.754366 (PMC8850939; doi:10.3389/fphar.2021.754366)
Supplement: Supplementary file 1 [file DataSheet1.PDF]

Supplementary Material

Supplementary Figure S1. Cost-effectiveness acceptability curve

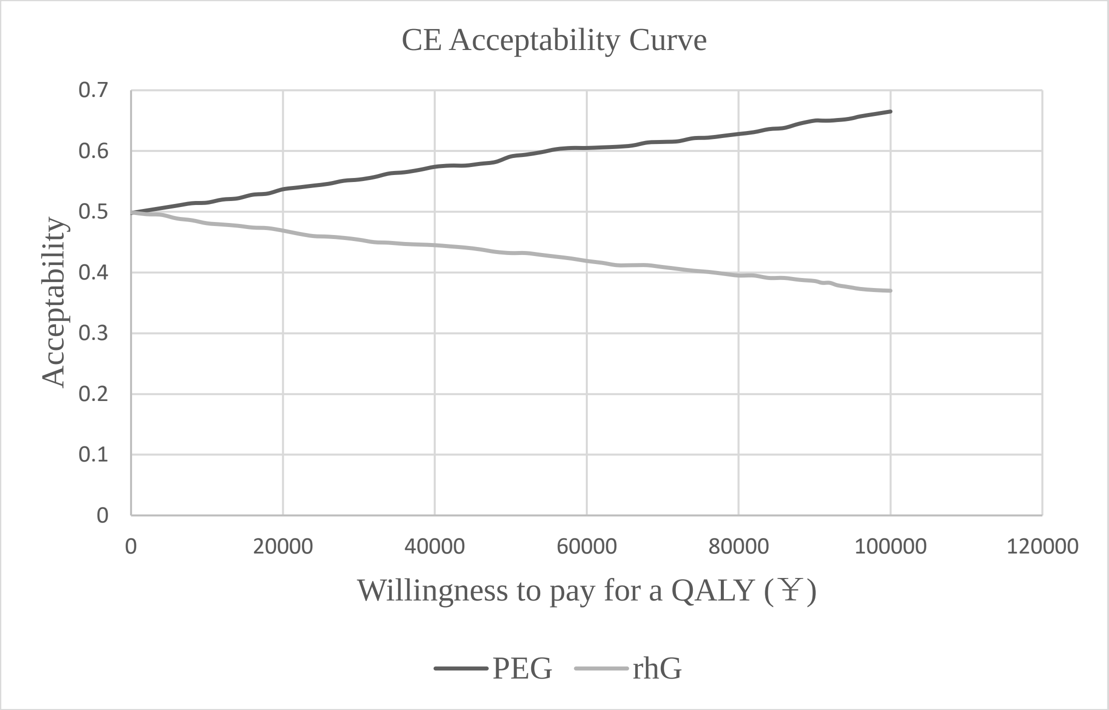

Supplementary Table S1. Results of the PSA for chemotherapy and post-chemotherapy models

	**PEG-rhG-CSF**	**rhG-CSF**	**95%CI**
Model one			
Cost	￥146148	￥145718	￥770-￥2865
QALYs	3.422	3.352	0.024-0.089
Model two			
QALYs	11.705	11.526	——
